# ﻿A new species of holothuroid from the Labrador Sea (eastern Canada): *Pseudothyonelabradorensis* sp. nov. (Echinodermata, Holothuroidea, Dendrochirotida, Sclerodactylidae)

**DOI:** 10.3897/zookeys.1206.123364

**Published:** 2024-07-08

**Authors:** Antonina Kremenetskaia, Tom Alvestad, Heather D. Penney, Jean-François Hamel, Bárbara de Moura Neves, David Côté, Annie Mercier

**Affiliations:** 1 Shirshov Institute of Oceanology, Russian Academy of Sciences, Moscow, Russia Shirshov Institute of Oceanology, Russian Academy of Sciences Moscow Russia; 2 Department of Natural History, University Museum of Bergen, University of Bergen, Bergen, Norway University Museum of Bergen Bergen Norway; 3 Department of Ocean Sciences, Memorial University, St. John’s, Newfoundland and Labrador, Canada Memorial University St. John’s Canada; 4 Aquatic Resources Program, St. Francis Xavier University, Antigonish, Nova Scotia, Canada St. Francis Xavier University Antigonish Canada; 5 Society for the Exploration and Valuing of the Environment (SEVE), St. Philips, Newfoundland and Labrador, Canada Society for the Exploration and Valuing of the Environment St. Philips Canada; 6 Northwest Atlantic Fisheries Centre, Fisheries and Oceans Canada, St. John’s, Newfoundland and Labrador, Canada Northwest Atlantic Fisheries Centre, Fisheries and Oceans Canada St. John’s Canada

**Keywords:** Bathyal fauna, distribution, Northwest Atlantic fauna, sea cucumbers, taxonomy

## Abstract

A new species of holothuroid, *Pseudothyonelabradorensis***sp. nov.** (order Dendrochirotida and family Sclerodactylidae), was discovered off the coast of Labrador (eastern Canada) at a depth of 740–969 m. Two specimens were described based on morphological and genetic parameters. Distinctive characters included pinkish body colour, presence of tube feet on a ‘tail’, supporting rod-shaped ossicles in the tube feet, and rod-shaped ossicles in the tentacles. To investigate its phylogenetic relationships, partial sequences of COI were obtained for the new species as well as for the type species *P.raphanus* and another North Atlantic species *P.serrifera.* According to the phylogenetic analysis, *P.labradorensis***sp. nov.** appeared in a well-supported clade with *P.raphanus* and *P.serrifera*. Molecular data also suggest polyphyly of the genus, showing the Northeast Pacific species *Pseudothyonebelli* recovered outside of the clade containing the type species. *Pseudothyonelabradorensis***sp. nov.** is the first species of the genus from the Northwest Atlantic. A key to the North Atlantic *Pseudothyone* is provided.

## ﻿Introduction

The genus *Pseudothyone* was established by Panning (1949) for *Thyoneraphanus* Düben & Koren, 1846 within the new subfamily Sclerodactylinae. Apart from the type species, Panning assigned five other species to this genus: *P.belli* (Ludwig 1886), *P.mosaica* (Koehler & Vaney, 1910), *P.poucheti* (Barrois, 1882), *P.argillacea* (Sluiter, 1910), *P.buccalis* (Stimpson, 1855) and *P.trachyplaca* (Clark, 1924). The latter two species were later assigned to other genera, whereas *P.poucheti* was synonymized with *P.raphanus* according to Théel (1886), and *P.argillacea* with *P.belli* according to Deichmann (1954).

*Pseudothyone* currently includes seven species characterized by a wide range of morphological characters. The original diagnosis by Panning (1949) included the following: ten tentacles, undivided pieces of calcareous ring and its radial pieces with two fork-shaped processes of medium length undivided or consisting of few large pieces, and body-wall ossicles composed of only plates. Later investigations of this genus added more variations to taxonomic characters (Lambert and Oliver 2001; Martins 2019): tentacles ten equal-sized (in *P.levini* Lambert & Oliver, 2001) or eight bigger and two ventral smaller in all other species; body-wall ossicles smooth plates and knobbed buttons (*P.belli*), or smooth plates only (all other species); tube feet ossicles tables and end plates (*P.belli* and *P.mosaica*), rods and end plates (*P.sculponea* Cherbonnier, 1958, *P.serrifera* (Östergren, 1898) and *P.levini*), or end plates only (*P.raphanus* and *P.furnestini* Cherbonnier, 1969); radial pieces of calcareous ring with short posterior processes (*P.levini*), or of medium length (all other species), with posterior processes undivided (*P.raphanus*), or divided into several pieces (*P.belli*, *P.mosaica*, *P.sculponea*).

**Figure 1.**
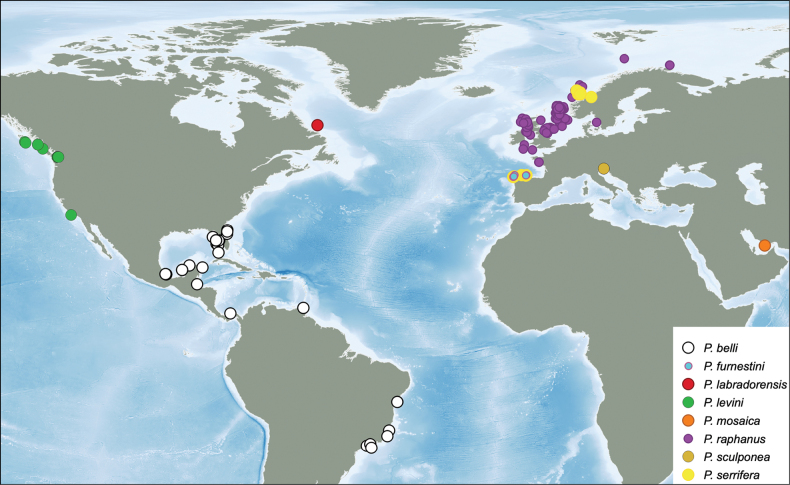
Distribution of *Pseudothyonelabradorensis* sp. nov. and other species in the same genus, based on published records and GBIF (2023). Map was prepared using *QGIS* 3.16.5-Hannover.

Most species of *Pseudothyone* are distributed in the Atlantic Ocean (Fig. 1). *Pseudothyonefurnestini*, *P.raphanus*, and *P.serrifera* occur in the Northeast Atlantic, and the latter two are also known from the Mediterranean. *Pseudothyonesculponea* is known only from the Mediterranean, and *P.belli* is from the Western Atlantic (Atlantic US coast, Caribbean and Brazil). Moreover, two species are known outside the Atlantic: *P.mosaica* from the Persian Gulf, and *P.levini* from the northeastern Pacific. Bathymetric distribution also differs between the species. The shallowest is *P.belli* occurring at sublittoral depths from the low-tide mark to 37 m (Pawson et al. 2010). Among shallow-water representatives are also *P.levini* occurring from the intertidal to 70 m depth and *P.sculponea* reported from 21 to 41 m (GBIF.org 2023). *Pseudothyoneraphanus* and *P.serrifera* occur deeper, at 10–1200 m and 200–1200 m, respectively (Madsen and Hansen 1994; Fernández-Rodríguez et al. 2019). The deepest-dwelling species is *P.furnestini* reported from 440–1347 m (Fernández-Rodríguez et al. 2019). *Pseudothyonemosaica* is known from a single record at 97 m.

Phylogenetic relationships of species within the genus *Pseudothyone*, as well as the latter’s position within Sclerodactylidae, remain unclear. The taxon was originally described by Panning (1949) as Sclerodactylinae, a subfamily of Cucumariidae. Later, Pawson and Fell (1965) upgraded its status to the family Sclerodactylidae with two subfamilies, Sclerodactylinae and Cladolabinae. Thandar (1989) restricted the diagnosis of Sclerodactylidae and described a new subfamily, Sclerothyoninae. Smirnov (2012) recognized the subfamilies Cladolabinae and Sclerothyoninae as separate families. Available molecular data (Miller et al. 2017) partly support the system of Smirnov, showing no sister relationships between sclerothyonins *Afrocucumisafricana* (Semper, 1867) and *Euthyonidiellahuwi* O’Loughlin in O’Loughlin et al., 2014 and sclerodactylins *Pachythyonerubra* (Clark, 1901) and *Sclerodactylabriareus* (Lesueur, 1824).

In this study we describe a new species, *Pseudothyonelabradorensis* sp. nov., from the bathyal depths of the Labrador Sea (Northwest Atlantic Ocean) based on morphological and molecular data. Using molecular data on partial sequences of the mitochondrial gene cytochrome *c* oxidase subunit I (COI), we examined phylogenetic relationships of *P.labradorensis* sp. nov. with two Atlantic congeners, *P.raphanus* (type species of the genus) and *P.serrifera*, for which we obtained additional genetic data, as well as with the Northeast Pacific species *P.levini*. COI is commonly used for recovering relationships within the genera of Holothuroidea (O’Loughlin et al. 2014; Li et al. 2018; Ogawa et al. 2022; Ogawa et al. 2023). To test monophyly of *Pseudothyone* we included available data on the representatives of Sclerodactylidae sensu Smirnov (2012) as well as *Pentameracalcigera* (Stimpson, 1851), as close relationships of *Pentamera* to Sclerodactylidae has been shown previously (Arndt et al. 1996; Miller et al. 2017).

## ﻿Material and methods

Two specimens of *Pseudothyonelabradorensis* sp. nov. were collected together in the same location (North Atlantic Fisheries Organization NAFO, Zone 2J https://www.marineregions.org/gazetteer.php?p=details&id=23382) using a rock dredge deployed during the ISECOLD scientific expedition aboard the research icebreaker CCGS *Amundsen* on 30 August 2020 (Table 1). The rock dredge net had a 7 mm mesh. Due to the large volume of mud collected at this station, a fourth of the material was sieved through a 2 mm sieve and the rest was scanned for larger individuals through a 17 mm diameter mesh sieve. The specimens later identified as *Pseudothyone* were preserved in 100% ethanol for morphological and molecular analysis.

**Table 1.** Voucher information for which molecular data were obtained in this study. Additional data can be obtained from corresponding BOLD Process ID pages.

Identification	Voucher	GenBank Acc.	BOLD Process ID	Catalog Number	Storing Institution*	Collection Date	Region	Latitude	Longitude	Depth (m)
*Pseudothyonelabradorensis* sp. nov.	AMLAB-02	PP047583	AMLAB002-21	ECH02801	IORAS	30/08/2020	coast of Labrador (eastern Canada)	56.500	-58.084	740–969
*Pseudothyonelabradorensis* sp. nov.	PT1210	PP047582	PSSP001-23	ECH02802	IORAS	30/08/2020	coast of Labrador (eastern Canada)	56.500	-58.084	740–969
*Pseudothyoneraphanus*	Sletvik2016_87	OR974836	ECHNO258-16	NTNU-VM-72185	NTNU	25/10/2016	Norway, Trondheimsfjorden	63.67830	9.79612	122
*Pseudothyoneraphanus*	ZMBN_120538	OR974833	ECHNO400-18	ZMBN 120538	ZMBN	08/06/2014	Norway, Halsnøyfjorden	59.75777	5.49778	60
*Pseudothyoneraphanus*	ZMBN_120547	OR974839	ECHNO409-18	ZMBN 120547	ZMBN	02/05/2006	Norway, Kobbaleia, Bergen area	60.314	5.156	22–42
*Pseudothyoneserrifera*	Sletvik2016_111	OR974842	ECHNO197-16	NTNU-VM-72204	NTNU	25/10/2016	Norway, Trondheimsleia	63.657	9.634	260–278
*Pseudothyoneserrifera*	Sletvik2016_88	OR974838	ECHNO259-16	ZMBN 155233	ZMBN	24/10/2016	Norway, Trondheimsfjorden	63.586	9.846	280–102
*Pseudothyoneserrifera*	Sletvik2016_89	OR974840	ECHNO260-16	NTNU-VM-72186	NTNU	24/10/2016	Norway, Trondheimsfjorden	63.586	9.846	280–102
*Pseudothyoneserrifera*	Sletvik2016_90	OR974837	ECHNO261-16	NTNU-VM-72187	NTNU	24/10/2016	Norway, Trondheimsfjorden	63.586	9.846	280–102
*Pseudothyoneserrifera*	Sletvik2016_91	OR974834	ECHNO262-16	NTNU-VM-72188	NTNU	25/10/2016	Norway, Trondheimsleia	63.657	9.634	260–278
*Pseudothyoneserrifera*	Sletvik2016_92	OR974835	ECHNO263-16	ZMBN 155234	ZMBN	25/10/2016	Norway, Trondheimsleia	63.657	9.634	260–278
*Pseudothyoneserrifera*	Sletvik2016_86	OR974841	ECHNO271-16	ZMBN 155235	ZMBN	26/10/2016	Norway, Trondheimsleia	63.594	9.508	56–45

*Institution abbreviations: IORAS: Shirshov Institute of Oceanology, Russian Academy of Sciences, Moscow, Russia NTNU: Department of Natural History, University Museum of Norwegian University of Science and Technology, Trondheim, Norway ZMBN: Natural History Collections, University Museum of Bergen, Bergen, Norway

The specimens of *P.raphanus* and *P.serrifera* had previously been collected in 2006–2016 with a Van Veen grab, Triangular dredge and Agassiz trawl deployed from the research vessels R/V *Gunnerus*, R/V *Håkon Mosby* and R/V *Hans Brattström* (Table 1). They were preserved and subsequently stored in 96% ethanol.

Morphological examination, dissection and photographing were performed using a Leica M205C stereomicroscope equipped with a Leica FLEXACAM C1 digital camera. To extract ossicles, small fragments of the body wall, introvert, tube foot and tentacle skin were digested in a domestic bleach water solution followed by several rinses in distilled water. For light microscopy, ossicles were transferred onto a glass slide, dried using a heating stage and mounted in Canada Balsam. For scanning electronic microscopy (SEM), ossicles were dried with 96% ethanol, mounted on a stub and sputter coated with gold. Ossicles were examined and photographed under a light microscope (Olympus BX43) with a ToupCam U3CMOS08500KPA digital camera and SEM examination was performed using a TESCAN Vega 3.

To evaluate phylogenetic relationships of *Pseudothyonelabradorensis* sp. nov. within the genus, partial sequences of cytochrome *c* oxidase, subunit I (COI) were obtained from two examined specimens. Additionally, COI data were obtained for three specimens of *P.raphanus* and seven specimens of *P.serrifera* (Table 1). Some of these specimens were collected close to their type localities (Bergen area and Trondheimsfjorden, respectively). Also, three sequences of *Pseudothyone*, publicly available in GenBank, were used in the analysis: *P.raphanus* (MG934913) and *P.levini* (MH242951 and MH242950). To test monophyly of *Pseudothyone*, published sequences of Sclerodactylidae sensu Smirnov (2012) were analysed (Table 2). A COI sequence of *Pentameracalcigera* (Phyllophoridae) was also included in the analysis as it was recovered in a sister clade to the sclerodactylid *Pachythyonerubra* (Miller et al. 2017). *Stichopushorrens* was set as an outgroup. GenBank accession numbers of the sequences used in the analysis are listed in the Table 2.

**Table 2.** Data on sequences obtained from Genbank.

Identification	Current family attribution	Voucher	GenBank Acc.
*Eupentactaquinquesemita*	Sclerodactylidae	BIOUG<CAN>:BAM00127	HM542177
*Eupentactaquinquesemita*	Sclerodactylidae	no data	U32218
*Eupentactapseudoquinquesemita*	Sclerodactylidae	BMBM-0776	MH242754
*Eupentacta* sp.	Sclerodactylidae	ECHINO_001_091	MK037199
*Eupentacta* sp.	Sclerodactylidae	USNM:IZ:1503386	MZ580563
*Eupentacta* sp.*	Sclerodactylidae	isolate CZ1	MK562383
*Havelockia* sp.	Sclerodactylidae	NMV_F151829	KF142167
*Pachythyonerubra*	Sclerodactylidae	SIO:BIC:E6676	KX874387
*Pentameracalcigera*	Phyllophoridae	BIOUG<CAN>:HLC-30032	HM543053
*Pseudothyonelevini*	Sclerodactylidae	BFHL-1914	MH242950
*Pseudothyonelevini*	Sclerodactylidae	BMBM-1095	MH242951
*Pseudothyoneraphanus*	Sclerodactylidae	Echin 6852V	MG934913
*Sclerodactylabriareus*	Sclerodactylidae	SIO:BIC:E6814	KX874342
*Stichopushorrens*	Stichopodidae	isolate SHP3	KY986418

*Identification according Turanov et al. (2024)

Molecular work was carried out in two laboratories applying two different protocols. Genetic data on *Pseudothyonelabradorensis* sp. nov. (voucher AMLAB-02), *P.raphanus* and *P.serrifera* were obtained at the Canadian Centre for DNA Barcoding, University of Guelph following protocols by Ivanova et al. (2006), Ratnasingham and Hebert (2007), and deWaard et al. (2008). Data on another specimen of *P.labradorensis* sp. nov. (voucher PT1210) was generated at IORAS using the following methods: DNA was extracted using QuickExtract^TM^ DNA Extraction Solution (Lucigen) following the manufacturer protocol; PCR amplification was conducted using Encyclo Plus PCR kit (Evrogen, Moscow) according the manufacturer protocol with annealing temperature set at 48 °C; PCR products were purified from agarose gel using HiPure Gel DNA Mini Kit (Magen); the purified samples were sequenced using the Sanger method on Applied Biosystems ABI 3900 (ThermoFisher Scientific) by Evrogen (Moscow, Russia). All PCR amplifications and sequencing were carried out using the LCOech1aF1 (5′-TTTTTTCTACTAAACACAAGGATATTGG-3′; D. Eernisse unpublished) and HCO2198 (5′-TAAACTTCAGGGTGACCAAAAAATCA-3′; Folmer et al. 1994) primers.

Contigs were assembled from forward and reverse chromatograms using the MUSCLE algorithm implemented in Geneious v.10.0.9 and then manually edited. Sequences were aligned in MEGA 7 (Kumar et al. 2016) also using the MUSCLE algorithm, and then checked for stop-codon presence. The final dataset included 840 aligned positions. Phylogenetic analysis was performed using maximum-likelihood (ML) and Bayesian inference (BI) approaches. PartitionFinder 2 (Lanfear et al. 2017) was used for selecting best-fit partitioning schemes and models of nucleotide evolution. The defined models were TRNEF+I+G for positions 1 and 2, and GTR+G for position 3. ML tree search and bootstrapping was conducted in RAxML-NG (Kozlov et al. 2019) using auto MRE option with cutoff=0.03; the analysis converged after 12950 replicates. BI analysis was performed using MrBayes v.3.2 (Ronquist et al. 2012). The analysis was conducted in two runs, four chains (one cold and three heated) with trees and parameters sampled every 500 generations. The traces were analysed in Tracer v.1.7.1, and then 10% of the trees were discarded as burn-in. Run convergence was evaluated by analysing sump output parameters in MrBayes *log* file and Tracer v.1.7.1. Genetic distances were calculated using Kimura 2-parameter model (K2P; Kimura 1980) implemented in MEGA 7.

## ﻿Taxonomy

### ﻿Order Dendrochirotida Grube, 1840

**Family Sclerodactylidae Panning, 1949 sensu Smirnov, 2012**

***Pseudothyone* Panning, 1949**

#### ﻿ Pseudothyone labradorensis sp. nov.

Taxon classificationAnimaliaDendrochirotidaSclerodactylidae

9B64AAFE-BD53-5697-8C2A-D3FE095FFB90

https://www.zoobank.org/B4110DB7-0589-4A96-BC32-344F56ABF794

Figs 2 , 3

##### Type material.

***Holotype*.** Canada • 9.5 mm in length; Labrador Sea, 56.500, -58.084, depth 740–969 m (between bottom and recovery); 30 Aug. 2020; Station ISECOLD -0-1000; rock dredge; substratum primarily mud with sparse rocks; IORAS ECH02801, ID AMLAB-02. ***Paratype*.** Canada • 14 mm in length, collected at same time and locality as holotype; IORAS ECH02802, ID PT1210. Both holotype and paratype are preserved and stored in 96% ethanol.

##### Diagnosis.

Body curved, cylindrical, tapered at anterior and posterior ends. Body colour in vivo pinkish. Tentacles 10, two ventral tentacles smaller. Tube feet arranged in several rows along radii, also present in interradii and on tapered posterior part of body. Body-wall ossicles slightly concave plates of irregular shape with smooth margins and perforations. Ossicles of tube feet rod-shaped; terminal plate irregular in shape; rods not numerous, smooth, with enlarged tuberculous ends. Tentacle ossicles rods with enlarged perforated ends. Segments of calcareous ring with undivided posterior projections.

##### Description.

**Figure 2.**
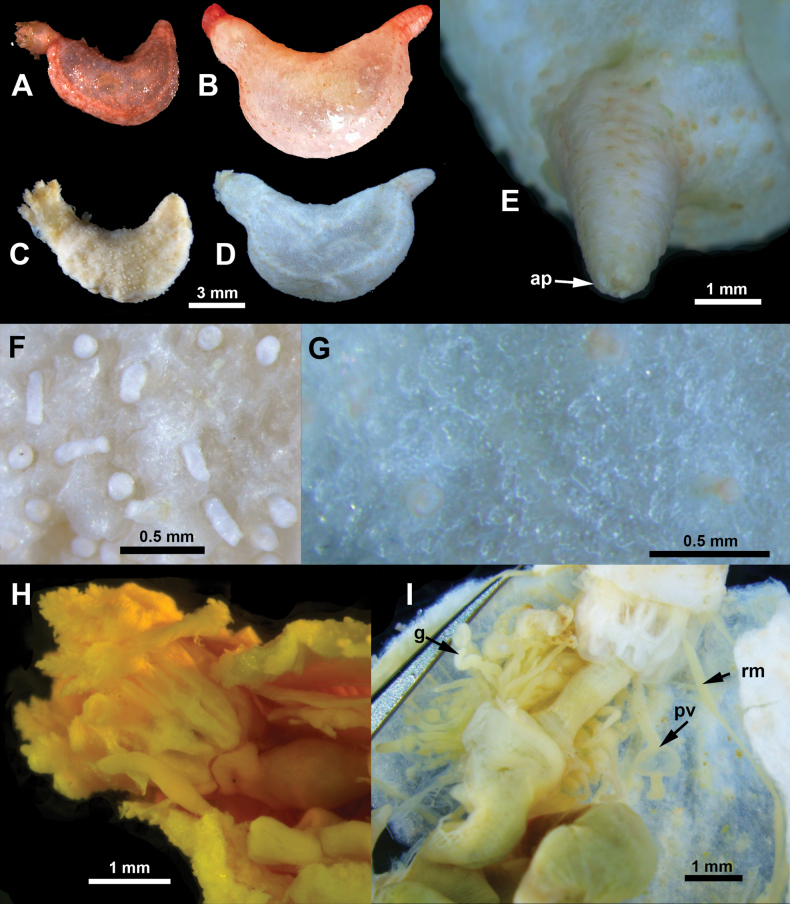
*Pseudothyonelabradorensis* sp. nov., external and internal morphology **A, C** holotype, before preservation (**A**) and preserved in ethanol (**C**) **B, D** paratype, before preservation (**B)**, in ethanol (**D**) **E** tapered posterior body part in paratype **F** tube feet in holotype **G** body-wall skin with dense layer of ossicles **H** segments of calcareous ring in holotype **I** dissected anterior part in paratype. Labels: ap anal papillae, g gonad, pv Polian vesicle, rm retractor muscle.

Body curved towards dorsal side, wider and cylindrical in the middle, anterior end tapered towards terminal mouth, posterior end tapered to a ‘tail’ towards terminal anus, ‘tail’ short, more prominent in paratype and short in holotype (Fig. 2A–D). Body colour pinkish in living specimens (Fig. 2A, B), tentacles, tube feet and ‘tail’ more brightly coloured; colour in ethanol uniformly greyish, tentacles and tube feet whitish or greyish (Fig. 2C, D). Body length 9.5 mm in holotype, 14 mm in paratype. Body-wall skin thin, rough, non-transparent, with dense layer of scale-like ossicles (Fig. 2G). Tentacles (examined in holotype, in paratype they were partly retracted) ten, two ventral tentacles remarkably smaller in size. Tube feet small (Fig. 2F), non-transparent in ethanol, arranged in several rows along radii and also scattered in interradii; tube feet more numerous on mid body, along ventrolateral and mid-ventral radii; on ‘tail’ tube feet smaller and less numerous, arranged in double rows (Fig. 2E). Anal papillae five (Fig. 2E). Pieces of calcareous ring up to 1.6 mm in length, united at most their length, radial segments with undivided posterior projections, with forked grooved anterior projections; interradial segments with grooved anterior projections (Fig. 2H, I). Retractor muscles undivided, not flat, broader anteriorly (Fig. 2I). Polian vesicle single, non-divided (Fig. 2I). Gonad in a tuft (Fig. 2I), gonad tubules in paratype with oocytes of different size.

**Figure 3.**
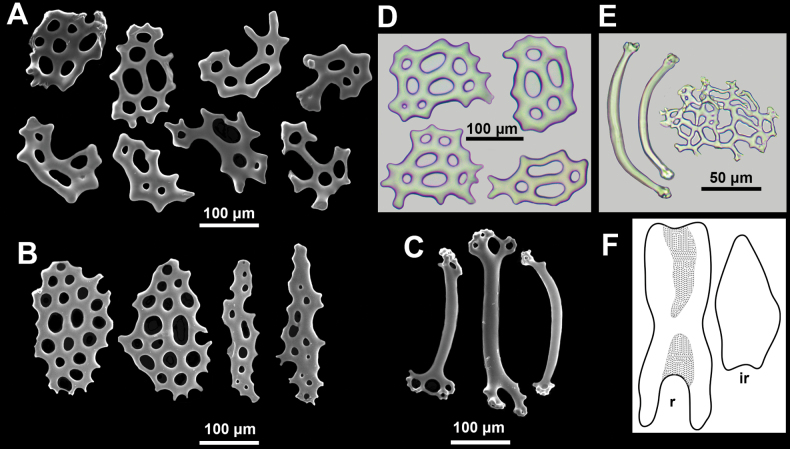
Ossicles of *Pseudothyonelabradorensis* sp. nov. **A, D** body-wall perforated plates **B** introvert perforated plates **C** tentacle rods **E** tube foot rods and terminal plate **F** right dorsal radial (r) and interradial (ir) segments of calcareous ring. **A, B, C**SEM images **D, E** light microscope images **F** drawing.

Body-wall ossicles in a single dense overlapping layer, laying their concave side out; body-wall ossicles small perforated plates, usually do not exceed 200 µm in length, slightly concave, mostly irregular in shape, smooth; perforations with smooth margins, from rounded to oblong in shape, their size and shape can vary even on a same plate (Fig. 3A, D). Introvert with bigger flat plates, plates often narrow and elongated (Fig. 3B). On tube feet supporting rod-shaped ossicles, not numerous, curved in shape, with slightly enlarged perforated ends, length 100–130 µm; terminal plate delicate, irregular in shape, ~70 µm in diameter (Fig. 3E). Tentacle ossicles curved rods, most ossicles ranged 100–480 µm in length, bigger in proximal part of tentacles; smaller rods sometimes enlarged in the middle; bigger rods often with enlarged spatulated and/or bifurcated ends (Fig. 3C).

##### Differential diagnosis.

*Pseudothyonelabradorensis* sp. nov. can be distinguished from other species by a combination of the following characters: pinkish body colour (may disappear after preservation in ethanol); tube feet arranged in double rows on tapered posterior end; body-wall ossicles only perforated plates with smooth perforation margins; introvert ossicles perforated plates; tube foot ossicles rods and terminal plate of irregular shape.

##### Distribution.

Canada, Labrador Sea, depth 740–969 m.

##### Etymology.

The species is named after its type locality.

### ﻿Environmental information

A CTD cast and drop camera deployment took place at 494 and 218 m respectively from the rock dredge recovery location. Bottom water temperature at 990 m was 3.7 °C, salinity 34.8 psu. These conditions are associated with Labrador Sea Water, and are in contrast to colder and less saline conditions found on the adjacent continental shelf (Côté et al. 2019). Modelled bottom water velocities (GIOPS daily historical 3D data extracted from https://navigator.oceansdata.ca/public/ for 2022) for this collection location average 0.13 m s^-1^ (range: 0.02–0.23 m s^-1^). Other fauna collected in the rock dredge deployment included soft corals *Duvaflorida* (Rathke, 1806), sea pens (Pennatuloidea), fragments of the gorgonians *Primnoaresedaeformis* (Gunnerus, 1763), *Paragorgiaarborea* (Linnaeus, 1758), *Acanellaarbuscula* (Johnson, 1862), and the mushroom coral *Anthomastus* Verrill, 1878. Sponges, bivalves, and a small grenadier were also present, highlighting a diverse range of bottom type requirements. The seafloor imagery further corroborates the dominance of soft sediment in the study area, with sparse gravel and rocks. Based on 42 seafloor images (along a 1.1 km long transect), 98% of the primary sediment was classified as fine sediment, and only 2.4% as gravel.

### ﻿Molecular data

**Figure 4.**
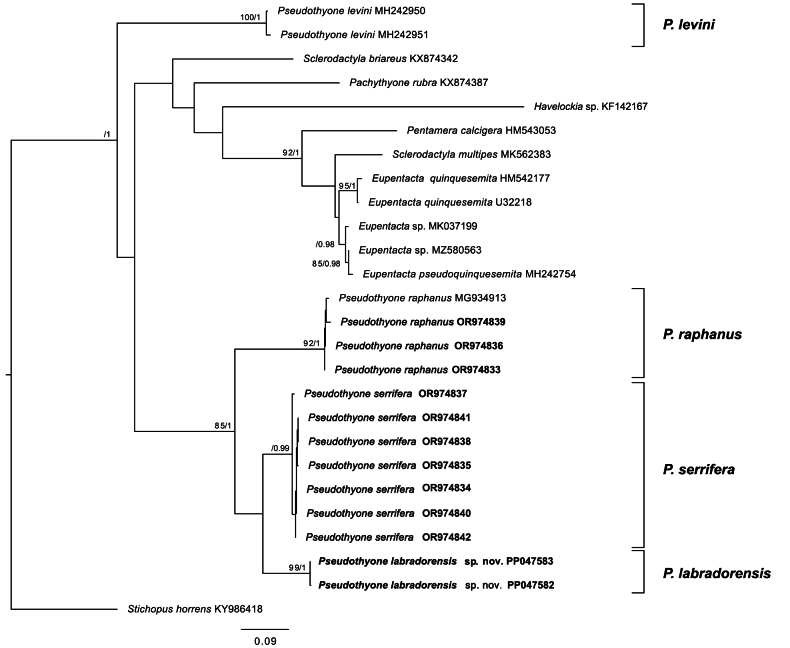
Phylogenetic relationships inferred using maximum-likelihood (ML) and Bayesian (BI) analyses of the COI dataset (840 bp). Topology corresponds to best-scoring ML tree; node values are bootstrap scores (≥75%) / BI posterior probabilities (≥0.95). Taxa examined in this study are in bold. *Stichopushorrens*KY986418 was set as outgroup.

Both examined specimens of *Pseudothyonelabradorensis* sp. nov. formed a single, well-supported clade [bootstrap (BS) 99, posterior probability (PP 1), Fig. 4]. The sequences of the holotype and paratype were assigned to the same Barcode Index Number (BIN, AEH8268) and showed little genetic divergence (K2P-distance 0.003). Based on the COI data, the closest taxa to *P.labradorensis* sp. nov. were *P.serrifera* (K2P-distance 0.10) and *P.raphanus* (K2P-distance 0.162). Phylogenetic analysis recovered *P.labradorensis* sp. nov. in a sister clade with *P.serrifera*, although this clade was weakly supported (BS 50, PP 0.84). A clade of *P.raphanus*, *P.serrifera* and *P.labradorensis* sp. nov. (*raphanus* clade) was well-supported in the BI analysis and averagely supported by ML (BS 85, PP 1). *Pseudothyonelevini* was sister to the clade with all other species of the dataset showing no close relationships with the *raphanus* clade nor with other examined sclerodactylids. The analysis showed no close relationships of the *raphanus* clade with examined sclerodactylid representatives.

## ﻿Discussion

**Figure 5.**
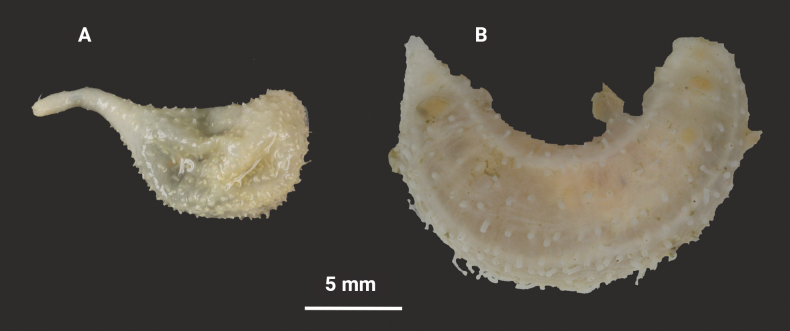
*Pseudothyoneraphanus* (**A**) and *P.serrifera* (**B**) collected from the type locality areas. **A**ZMBN 120547 **B**NTNU-VM-72187. Collection data is given in Table 1. Image courtesy of Katrine Kongshavn.

Molecular data supported close relationships of *Pseudothyonelabradorensis* sp. nov. to the type species of the genus, *P.raphanus*, and to the Northeast Atlantic species *P.serrifera.* According to body-wall ossicle morphology, *P.labradorensis* sp. nov. is most closely related to *P.raphanus* (Fig. 5A), from which it differs by its pinkish body colour, presence of tube feet on the ‘tail’, absence of fern-like ossicles on the tentacles, and by the presence of rod-shaped ossicles in the tube feet. From the genetically closest species, *P.serrifera* (Fig. 5B), the new species differs by the pinkish body colour, absence of S-shaped rods on tentacles, and by the smooth margins and perforations of body-wall ossicles. From another Northeast Atlantic species, *P.furnestini*, the new species differs by body colour and body-wall ossicles. *Pseudothyonefurnestini* is characterized by whitish colouration and thick body-wall ossicles that are from rounded to oval in shape and often possess solid, unperforated extensions. Also *Pseudothyonefurnestini* lacks rod-shaped ossicles in the tube feet.

According to molecular data, *Pseudothyonelevini* was not closely related to the species of the *raphanus* clade. This species has ten tentacles that are equal in size, whereas other species of *Pseudothyone* have two ventral tentacles smaller than others. From species of the *raphanus* clade, it also differs by a less prominent ‘tail’, less perforated body-wall plates and by more robust rod-shaped ossicles on tube feet.

Some other species of *Pseudothyone* also have remarkable morphological differences. Apart from plates, the body-wall ossicles of *P.belli* include knobbed buttons and plates with handles, which do not occur in *P.raphanus* and most other species of the genus. Also *P.belli* differs by the ossicles from the tentacles, which are plates and rosettes (not rods as in *P.raphanus* and other species), by introvert ossicles pillared tables and plates, and by tube feet ossicles arched pillared tables. Therefore, *P.belli* differs remarkably from *P.raphanus* in most ossicle types. Marked differences in ossicle types are also noted for *P.mosaica*. This species is characterized by large rounded plates on the body wall and arched pillared tables on its tube feet.

The present results suggest that the taxonomy of *Pseudothyone* requires further investigation. Particularly, the generic affiliation of *P.levini*, *P.belli* and *P.mosaica* may require additional evaluation. The taxonomic position of the genus also remains unclear. Based on COI data, the *raphanus* clade (*P.raphanus* + *P.serrifera* + *P.labradorensis* sp. nov.) does not form any well-supported clade with other examined representatives of Sclerodactylidae sensu Smirnov (2012). More data, both morphological and molecular, are needed to analyze the phylogenetic relationships of *Pseudothyone*.

Scientific collections of fauna in the Labrador Sea are extremely limited so the distribution of this species is yet to be resolved. *Pseudothyonelabradorensis* sp. nov. is known from a single locality in the Labrador Sea, at a depth between 740–969 m. Metabarcoding surveys of the ISECOLD transects in the Labrador Sea (Côté et al. 2023) did not detect this species but several other holothuroids were detected over benthic habitats at a depth of 1026 m about 300 km to the north (Côté unpubl. data): *Chiridotalaevis* (O. Fabricius, 1780), *Benthogone* sp., *Enypniastes* sp., Psolidae gen. sp., and two unresolved species from the orders Dendrochirotida and Apodida.

Apart from *P.labradorensis* sp. nov., three more species of *Pseudothyone* are known from bathyal depths. The type species *P.raphanus* and another Northeast Atlantic species, *P.serrifera*, were reported down to 1200 m (Fernández-Rodríguez et al. 2019), with most of their records obtained shallower than 250 m and 600 m, respectively. The deepest species of the genus, *P.furnestini*, occurred between 440 and 1347 m (ibid.). According to McKenzie (1991), the body colour in *P.raphanus* can be “sometimes a pale pink”. Combined with personal unpublished observations of pinkish specimens of P.cf.raphanus off south Iceland, it is possible that *P.labradorensis* sp. nov. may have a wider distribution in the North Atlantic.

### ﻿Key to the North Atlantic *Pseudothyone*

**Table:** 

1	Tube feet present on tapered posterior part of body	**3**
–	Tube feet absent or few on tapered posterior part of body	**2**
2	Body-wall ossicles surrounding tube feet with elongated non-perforated prolongations; terminal plate in tube feet star-shaped	***P.sculponea***
–	Body-wall ossicles lack prolongations; terminal plate of irregular shape or absent	***P.raphanus***
3	Body-wall ossicles smooth plates and knobbed buttons	***P.belli***
–	Body-wall ossicles smooth plates only	**4**
4	Tube feet ossicle terminal plate only	***P.furnestini***
–	Tube feet with supporting rods and terminal plate	**5**
5	Body-wall plate perforations with serrated margins	***P.serrifera***
–	Body-wall plate perforations smooth	***P.labradorensis* sp. nov.**

## Supplementary Material

XML Treatment for Pseudothyone labradorensis

## ﻿Additional information

### Sampling and field studies

Permission for the field work and sample collections were obtained by the authors from the Department of Fisheries and Oceans Canada.

### Author ORCIDs

Antonina Kremenetskaia https://orcid.org/0000-0001-8851-3318

Tom Alvestad https://orcid.org/0000-0003-0204-2936

Heather Penney https://orcid.org/0000-0001-7259-3931

Jean-François Hamel https://orcid.org/0000-0002-0435-4127

Bárbara de Moura Neves https://orcid.org/0000-0003-2316-9223

David Côté https://orcid.org/0000-0002-5805-5077

Annie Mercier https://orcid.org/0000-0003-0807-2378

### Data availability

All data generated during this study are included in this article. Sequences are published in GenBank and BOLD.
